# Editorial: Central Nervous System Metastases in Lung Cancer Patients: From Prevention to Diagnosis and Treatment

**DOI:** 10.3389/fonc.2018.00511

**Published:** 2018-11-06

**Authors:** Lizza E. L. Hendriks, Deepa S. Subramaniam, Anne-Marie C. Dingemans

**Affiliations:** ^1^Department of Pulmonary Diseases, GROW School for Oncology and Developmental Biology, Maastricht University, Maastricht, Netherlands; ^2^Division of Hematology-Oncology, Georgetown University, Washington, DC, United States

**Keywords:** brain metastases from lung cancer, leptomeningeal metastases, treatment—contemporary views, diagnosis, driver mutations, cranial irradiation

## Introduction

Approximately 40% of lung cancer patients will develop central nervous system (CNS) metastases during the course of their disease (1). Most of these are brain metastases (BM), but 3–9% will develop leptomeningeal metastases (2, 3). The proportion of lung cancer patients diagnosed with CNS metastases has increased over the years due to increased use of brain imaging as part of initial cancer staging, advances in imaging techniques and better systemic disease control (4–6). CNS metastases can have a negative impact on quality of life (QoL) and overall survival (OS) (7–9). As such, prevention of CNS metastases development, as well as optimal treatment of already established CNS metastases is important.

Contributors in this Research Topic of Frontiers in Oncology, section Thoracic Oncology describe the advances in CNS metastases management in lung cancer patients, from prediction and prevention, to diagnosis and treatment.

## Prediction and prevention of cns metastases development and diagnostic pitfalls

Known risk factors for CNS metastases development are small cell lung cancer, adenocarcinoma histology, advanced nodal status, tumor stage and younger age (10–13). Patients with a driver mutation have a high risk of CNS metastases (14). This seems mainly due to their long survival, combined with the poor blood-brain-barrier penetration of the older generation tyrosine kinase inhibitors (TKIs) (Pedrosa et al.). However, these factors alone cannot predict accurately enough which patients will develop CNS metastases and better prediction models are needed. In the review of Pedrosa et al. the process of BM development and the evidence for the clinical and molecular factors associated with increased risk of BM diagnosis in lung cancer is summarized. In addition, they provide an excellent overview of new promising strategies to identify patients at high risk for BM development, including signatures derived from circulating tumor DNA measurements, single-nucleotide polymorphisms, copy number alterations, microRNAs and long non-coding RNAs (Pedrosa et al.). As it is known that among the non-small cell lung cancer (NSCLC) patients treated with curative intent, stage III patients have the highest risk of brain metastases development [30% (9, 15, 16)], preventive treatments such as prophylactic cranial irradiation (PCI) have been studied especially in this patient population (15, 16). Most studies have been published before the year 2000, but afterwards three new trials incorporating adequate baseline brain imaging, have reported their results. All studies showed a reduction of BM incidence after PCI compared to no PCI (Witlox et al.). Other important factors to consider before administering PCI are toxicity (acute as well as long-term), QoL and OS. Witlox et al. provide an up-to-date systematic review and meta-analysis of all published studies in this field. They provide detailed data on the effects of PCI on BM reduction, toxicity, QoL and OS and discuss areas for future research.

Although brain imaging techniques have improved over the years facilitating the diagnosis of BM, differential diagnosis can be challenging, especially in a patient with a medical history of cancer, as is discussed in the case report by Vanfleteren et al. It is stressed that even in an immunocompetent patient, a diagnosis of cerebral aspergillosis cannot be excluded. A short review of existing literature on this topic is also provided.

## Local treatment of brain metastases

When BM are diagnosed, local treatment options consist of radiotherapy [stereotactic radiosurgery (SRS) or whole brain radiotherapy (WBRT)] and surgery (17, 18). SRS is more and more often used in the treatment of BM. In the past, SRS was mainly used for up to four BM, but a recent trial showed that SRS is feasible for a higher number of BM (19). More than fifty percent of BM patients treated with SRS will experience an intracranial relapse (19, 20), and especially in this palliative setting patient participation in the decision making around available treatment options [e.g., SRS, WBRT, systemic treatment (with/without concurrent cranial irradiation), best supportive care] is important. Hartgerink et al. discuss the current evidence of SRS for NSCLC BM, and the incorporation of decision aid tools in the future directions for NSCLC BM treatment. Furthermore, local treatment can be complicated by symptomatic radiation necrosis for which no high-level evidence-based treatment exists, although bevacizumab is a promising treatment option (21–23). Differential diagnosis of radiation necrosis and BM progression can be difficult (21, 22). Loganadane et al. provide a very nice overview of the pathobiology, epidemiology, predictive factors, diagnosis and emerging treatment of radiation necrosis, with a specific focus on NSCLC.

## Systemic treatment of cns metastases

Systemic treatment options for BM have expanded over the last years (24, 25). Until recently, chemotherapy was the only treatment option with a poor penetration in the CNS (26). Angiogenesis inhibitors are promising in the treatment of NSCLC BM (23, 27, 28), but clinical trials of anti-angiogenic agents in NSCLC have largely excluded BM patients (29). Furthermore, TKIs have improved prognosis significantly in those with a druggable driver mutation (25, 30, 31). Newer TKIs are often designed to have better CNS penetration compared to first-generation TKIs (24, 30, 31). In the review of Kelly et al. the management of CNS metastases in *EGFR* mutated NSCLC patients is discussed, including the role of newer generation EGFR-TKIs, immunotherapy, and EGFR-TKIs combined with cranial irradiation or angiogenesis inhibition. Remon and Besse provide a broader overview of incidence and treatment of BM in oncogene addicted NSCLC patients, including rare driver mutations such as *ALK, ROS1, RET, BRAF*, and *NTRK*. Relevant research questions such as optimal sequence of treatment (upfront cranial irradiation or upfront TKI, sequence of TKI) are also discussed. In the case report of Meedendorp et al. treatment of a BM patient with acquired resistance to EGFR-TKI is discussed, stressing the importance of a molecular tumor board for decision making. Last, leptomeningeal metastases remain challenging to treat, especially in non-oncogene addicted NSCLC patients. Turkaj et al. provide an up-to-date review of possible treatment options for these patients, including systemic as well as intrathecal chemotherapy as well as radiotherapy options.

## Conclusion

With the articles in this Research Topic, we hope to provide a review of present and future treatment options for lung cancer CNS metastases, including evidence for predictive markers, preventive treatments, and local as well as systemic treatment options for already diagnosed CNS metastases.

## Author contributions

All authors listed have made a substantial, direct and intellectual contribution to the work, and approved it for publication.

### Conflict of interest statement

LH: none related to current manuscript, outside of current manuscript: research funding Roche, Boehringer Ingelheim (both institution), advisory board: Boehringer, BMS, (both institution), travel reimbursement: Roche, BMS (self). DS: speaker's bureau Astra Zeneca, Takeda; advisory board Astra Zeneca, Takeda, BMS; institutional research funding as PI: BMS, Genentech, Incyte, Lilly Oncology, Astra Zeneca, Novocure. A-MD: none related to current manuscript, outside of current manuscript: advisory board BMS, MSD, Roche, Eli Lilly, Takeda, Pfizer, Boehringer Ingelheim (all institution).
